# Prevalence of Hallucinations in the General Croatian Population

**DOI:** 10.3390/ijerph18084237

**Published:** 2021-04-16

**Authors:** Jelinčić Ivana, Degmečić Dunja

**Affiliations:** 1 Faculty of Dental Medicine and Health, Josip Juraj Strossmayer University of Osijek, Crkvena 21, 31 000 Osijek, Croatia; ijelincic9@gmail.com; 2 Clinical Hospital Centre Osijek, Psychiatric Clinic, Josipa Huttlera 4, 31 000 Osijek, Croatia; 3 Faculty of Medicine, Josip Juraj Strossmayer University of Osijek, Josipa Huttlera 4, 31 000 Osijek, Croatia

**Keywords:** prevalence, hallucinations, general population, epidemiology

## Abstract

Background: Hallucinations involve sensing things such as visions, sounds, or smells that seem real but are not. These things are created by the mind. However, little is known about the distribution of incident hallucinations in the community. This paper aims to examine manifestation and frequency of the hallucinatory experiences within the general Croatian population. Methods: The instrument of the survey is Chicago Hallucination Assessment Tool (CHAT). The study included 521 examinees; 284 females (54.5%) and males 237 (45.5%). Results: There was a manifestation of all types of hallucinatory experiences determined. Out of all of the participants 17% listed that they experienced acoustic hallucinations during their lifetime, 15% said that they have experienced visual hallucinations, 12% olfactory hallucinations, 10% gustatory and 12% tactile/cenesthetic hallucinations. Conclusion: The results of this research have indicated that simple hallucinations were mostly represented among the general Croatian population and those more complex were represented less which is a positive thing because of its prominent clinical significance. The contribution of this study is the possibility of comparisons with studies from different regions of Europe and the world. This is another component in a better understanding of the incidence of hallucinations in the general population. The data we have obtained puts us on the map of countries trying to raise awareness of a topic that needs to acquire more attention.

## 1. Introduction

Hallucinations as experiences are as old as the human species and all the way until the 19th century these experiences were explained as something mystical or divine. Recently the hallucinations are treated as pathological conditions and signs of diseases [1]. American psychiatric association (APA) defines hallucinations as compelling sense of reality but that happens without the external stimulation of the corresponding sense organ. They can affect any sense organ or different sensory modalities in case of complex hallucinations [2]. Distorted perception should be considered as a symptom and not as a disease (3) because distorted perception can be a guideline for diagnosing other pathological conditions in a patient (head injury or a brain tumor). It doesn’t affect exclusively the sick people nor does it always indicate a disease [3]. Studies that used the self-evaluation and interview of the examinees as methods, have proved that the incidence is much frequent than originally thought; with meta-analysis the result is 5–8 % in general population. That is about ten times more than manifestation of other mental disorders [4]. Studies that included students, patients from primary health care and general population have shown that the psychotic experiences are surprisingly more common among more than 50% of the examinees [5,6]. Many results of various epidemiological studies on large population samples from USA, Australia, Netherlands and Great Britain have shown defined psychotic phenotypes in range of 5–28% [7,8,9,10,11,12]. There were extensive epidemiological surveys of psychotic experiences of people during the last decade conducted [13,14]. Those are hallucinations and delusions which appear in the spectrum of reality testing with symptoms of psychotic disorders but they don’t necessary reach the full level of a psychotic intensity. While the initial researches in this field are focused on exploring increased risk for psychotic disorder, the newer researches have shown that the hallucinations are also connected to affective, anxiety and behavioral disturbances [15,16]. Studies around the developed world have confirmed that the psychotic symptoms in society are connected to the characteristic symptomatology disorders, higher rate of using health care services and an increased rate of suicidal ideation [8,17]. This study aimed to to determine manifestation and frequency of the hallucinatory experiences within the general population in Osijek city in Eastern Croatia; the frequency of hallucinations in relation to the basic characteristics of the participants and the frequency of individual hallucinations according to the division into simple/complex and subjective / external impression. 

## 2. Materials and Methods

### 2.1. Participants

This study was conducted in the Osijek city in the Eastern Croatia. We sampled participants by a simple random sampling at primary and high school, local shopping center, sports club, nursing home and local factory (individuals without reference to any specific characteristic) so that the only inclusion criteria was: adults (age 18 years and older) who are willing to participate in a study. There were no exclusion criteria. Each participant was informed about the research and gave written consent. The study was approved by the Ethics Board of the Clinical Hospital Centre Osijek. Out of 700 participants, 521 were included (insufficient effort responding to surveys).

### 2.2. Measures

The instrument of the survey is Chicago Hallucination Assessment Tool (CHAT). The CHAT was first translated to Croatian, then back-translated to English and then the third translator compared the original and back-translated version and prepared the final draft. The questionnaire is composed of six categories that refer to types of hallucinations and each category has additional questions to verify the hallucinatory experiences so that it is determined if they should be additionally monitored: (visual (9 questions), acoustic (8 questions), olfactory (7 questions), tactile (8 questions), gustatory (7 questions), and an additional category that is related to hallucinations that aren’t listed in previously mentioned categories (1 question). We divided hallucinatory experiences as simple/complex and subjective /external impression: simple visual hallucinations (lights, colors, geometric shapes, and indiscrete objects) and complex visual hallucinations (clear, lifelike images or scenes). Simple acoustic hallucinations (hissing, whistling, an extended tone) and complex acoustic hallucionations (voices, music, or other sounds that may or may not be clear, may be familiar or completely unfamiliar, and friendly or aggressive). Whilst the term simple or complex has been used to classify hallucinations in the auditory and visual domains this distinction does not readily transfer to olfactory hallucinations beacuse most of them appear to be complex. Simple olfactory hallucinations can be described as an misperception of an odor which we cannot determine their origin. Most of the tactile/cenesthetics hallucinations also appear to be complex, except basic feeling of mispercepted itch on person’s skin. All categories of hallucinations are divided into subjective (participant experiences and reports hallucinatory experience) and external impression (persons around the participant notice the existence of an hallucinatory experience). The used questions are closed type of questions so the respondents answered with “yes” or “no.” There was also an adjusted demographic questionnaire used where the participants were filled in general information about themselves (gender, age, marital status, address, professional qualification and working status). To fulfill the questionnaire 10-15 min were needed and information were collected throughout a period from 01/03/2019. to 15/09/2019.

### 2.3. Statistical Analysis

Categorical data are represented by absolute and relative frequencies. Numerical data were described by the median and the limits of the interquartile range. Differences of categorical variables were tested by Chi-square Test and by Fisher’s exact test. The normality of the distribution of numerical variables was tested by the Shapiro-Wilk test. Differences between two independent groups were tested by Mann-Whitney’s U test. Differences in numerical variables in cases of 3 and more groups were tested by Kruskal-Wallis test (post hoc Conover). The significance level was set to Alpha = 0.05. MedCalc^®^ Statistical Software version 19.6 (MedCalc Software Ltd., Ostend, Belgium; https://www.medcalc.org accessed date: 30 January 2019) was used for statistical analysis.

## 3. Results

The research was performed on 521 respondents, median age is 32 (interquartile range from 25 to 45 years of age) in the range of 18 to 81 years of age. Female are 54.5% and male 45.5%. Married is 65% respondent, with median age that they spent in marriage- 14 (interquartile range from 5 to 27 years of marriage). Single/unmarried are 41%. There is a significant difference in marital status in relation to gender (χ^2^ test, *p* = 0.003). Women are significantly more often married while men are 108 unmarried (χ^2^ test, *p* = 0.003). In the city live 65% of the examinees and in their own 109 apartment or a house 53% of them, which are significantly more women (χ^2^ test, *p* = 0.02), considering the level of education, 68% of the examinees have high school education, and according to the working status, 67% of the examinees are employed (Table 1).

The frequency of auditory, visual, olfactory, sensory and taste hallucinations was checked through 35 questions, and each of the hallucinations was divided according to simple or complex hallucinations, and according to the impression on the subjective or external impression of hallucinations. The internal reliability of the whole Cronbach Alpha scale is 0.893, and of individual hallucinations from 0.658 to 0.763) (Table 2). 

Control of sound hallucinations is significantly needed more by respondents who live in their own house or apartment, compared to those who live with their parents (χ^2^ test, *p* = 0.006), or those who are employed compared to the unemployed (χ^2^ test, *p* = 0.006). Control of visible hallucinations is significantly more in need of men than in women (χ^2^ test, *p* = 0.04), which is significantly less in respondents living with parents compared to those living in their own condition or at home (χ^2^ test, *p* = 0.04). There are no significant differences in olfactory, sensitive or taste hallucinations in relation to the characteristics of the subjects (Table 3).

The frequency of complex olfactory hallucinations is significantly more common in men (Mann Whitney U test, *p* = 0.03), and the frequency of subjective impression of sound hallucinations is significantly higher in respondents living in the city (Mann Whitney U test, *p* = 0.03), while there are no significant differences in the type and impression of hallucinations according to other characteristics of the subjects.

**Figure 1:**
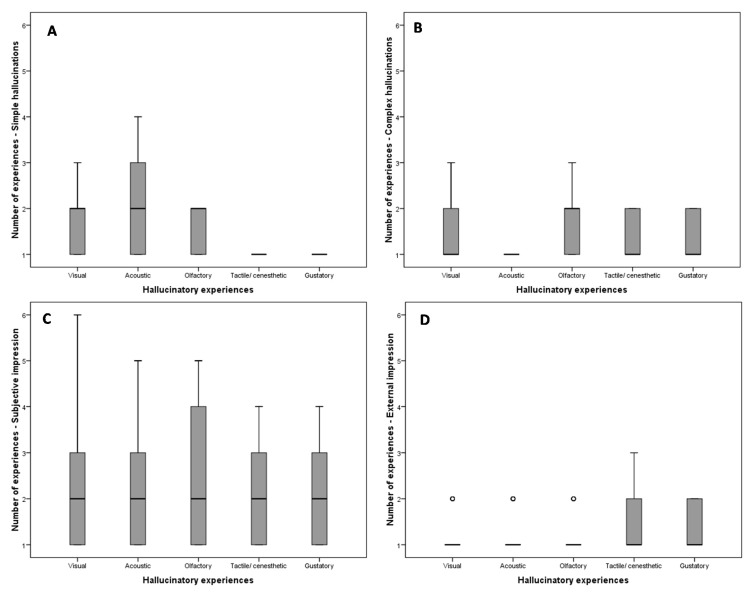
Frequency of individual hallucinations according to whether they are simple (**A**) or complex (**B**), or according to the impression: subjective (**C**) or external (**D**) (° = outliers).

Given the frequency of simple and complex hallucinations, and subjective and external impression, there is a significant difference according to the type of hallucination (Kruskal Wallis test, *p* < 0.001) (Figure 1).

The frequency of complex olfactory complications is significantly more common in men (Mann Whitney U test, *p* = 0.03), and the frequency of subjective impression of sound hallucinations is significantly higher in respondents living in the city (Mann Whitney U test, *p* = 0.03), while there are no significant differences in the type and impression of hallucinations according to other characteristics of the subjects (Table 4).

In relation to place of residence, the only significant difference is in the frequency of subjective impression of sound hallucinations (Kruskal Wallis test, *p* = 0.03), while in other hallucinations there are no significant differences according to place of residence and where they live (Table 5).

There are no significant differences in the frequency of simple and complex, and according to the impression of subjective and external hallucinations in relation to the level of education and employment status (Table 6).

## 4. Discussion

There are more and more evidence that prove the continuity of psychotic experiences like hallucinations in the general population. There are also many instruments developed that do the research of the hallucinatory experiences and many studies have discovered that the hallucinations are experienced by individuals from non-clinical sample of the population. Predictors of the clinical outcome for individuals with their hallucinatory experiences include their beliefs that they have had so far and their experiences, moods and subjective feel of control. These factors suggest key ideas for providing help to the people that have experiences the listed happenings [17]. The recent research show that the psychotic symptoms or experiences similar to psychotic experiences report not only patients with psychosis but also healthy members of the general population. It’s considered that healthy individuals that report these symptoms represent non-clinical phenotype of psychosis and it’s proved that they have an increased risk of schizophrenia [18]. The results of a large research about prevalence of psychotic symptoms in a form of visual or acoustic hallucinations have discovered that 12.7% of examinees have reported that they experienced one or more hallucinations during their lifetime. Many studies have questioned psychotic experiences within the general population around the developed world. Studies from the USA (NCS and NCS-R), Australia and Netherlands (NEMESIS) have established that the rate of psychotic symptoms around general population varies from 9–28%. Other studies that used a wider definition of psychotic symptoms have informed about different rates of hallucinations. British national research recorded hallucinations by 4.2% participants while the American NCS-R study has established that 9% of the distribution various psychotic symptoms in general population: mostly acoustic (6.4%) and visual hallucinations (4%) [19]. Causes for hallucinations can be multiple. Among the general population the most common reasons are: high fever, dehydration, disfunction of the thyroid gland, intensive negative emotions (stress, mourning, loss of a close one), loss of the sense of sight and hearing, brain tumors and extreme starvation. Hallucinations can occur also as a response to over-expectation related to a certain event or condition (for example, when a person is expecting an important call it’s possible to hear the phone ringing and that sound only happens to be a fruit of it’s imagination.) Manifestation of hallucinations among the general population can be observed from two aspects. First aspect is the division between simple and complex (those that can have clinical significance). Within the other aspect we divided them according the impression into: subjective and external impression. Acoustic hallucinations in this research are represented with 17% and visual with 15%. Affirmative answers by simple acoustic and visual hallucinations are, in relation to the research in total, expressed in the highest percentage. The examinees have answered affirmative when it comes to simple hallucinations in much larger number precisely because of the subjective impression of the examinee. On the contrary, the external impression of the hallucinations shows half as much affirmative answers. Complex acoustic and visual hallucinations have the same number of affirmative answers as the percentage of the manifestation among the general population in this research. The reason for high affirmation of the answers for further control of the prevalence acoustic (74.5%) and visual (64.9%) hallucinations are firstly because those examinees with simple hallucinations were included and because of their subjective impression about the existence of the hallucinations. There were seventeen studies from nine countries identified. Prevalence ranged from 0.6% to 84%. The differences in manifestation can partly exist because of the differences in definitions and methodologies but also variations based on gender, ethnicity and cultural surrounding. Some authors consider that only 1 out of 100 people have experienced acoustic hallucinations [17,20] while others consider that that number goes up to 71% [21]. High prevalence among the general population supports the idea that “hearing voices” isn’t per se necessary a pathological phenomenon, but that also distress connected to a negative experience and an inadequate coping could be connected to psychological disorder. A study that is specially designed to explore the frequency of acoustic hallucinations among the general population has, through a questionnaire interviewed 2.533 participants at the age of 18 and more. In total 7.3% of the samples have registered occurrence in their lifetime. As one of the limitations of the study is a lower percentage of fully fulfilled and accepted questionnaires (32.4%) [22]. Research has shown that more frequent check-ups are needed in people who have auditory and visual hallucinations and live alone in an apartment or house compared to those who live with their parents. The subjective impression of sound hallucinations is more frequent among respondents living in the city, due to the complex influences of the external environment: road noise, factory noise, high population density and traffic jams.

Occurrence of olfactory and tactile/cenesthetic hallucinations among the general population that has been obtained by this research is 12%. Affirmative answers within simple olfactory and tactile/cenesthetic hallucinations are represented in significantly larger number in relation to the occurrence of those hallucinations, precisely because of the subjective impression. External impression by olfactory and tactile/cenesthetic hallucinations shows less number of affirmative answers in relation to the occurrence among the general population. Complex olfactory and tactile/cenesthetic hallucinations are represented in larger number than the occurrence among the general population that can be explained with a wrong perception of the existing stimulus. High percentage of the examinees in this research shows that they should be furtherly controlled because of olfactory (61%) and tactile/cenesthetic (56.4%) hallucinations and that is because of including all of the examinees with simple hallucinations and their subjective impression of having them. Hallucinations are perceptual phenomena that are included in many parts of pathology. Although they are clinical widely researched, studies among the general population of these phenomena are scarce. This matter was researched by using the sample of the non-institutionalized general population of the United Kingdom, Germany and Italy. Participants were aged 15 and over. There were mental disorders and hallucinations explored (visual, acoustic, olfactory, haptic and gustatory hallucinations, out-of-body experiences, hypnagogic and hypnopompic hallucinations). In total, 38.7% of the examinees reported to have had a hallucinatory experience (19.6% less than once a month; 6.4% monthly; 2.7% once a week; 2.4% more than once a week) so from that we can conclude that the prevalence of the hallucinations among the general population isn’t negligible. It’s interesting that 8.6% of the examinees have reported to have olfactory hallucinations while 3.5% said that they experience them at least once a month [20].

The prevalence of the gustatory hallucinations among the general population, said by this study, is 10%. The simplest form of hallucination was presented by one experience that is multiply more prevalent in relation to the general prevalence. The examinees have answered affirmative in relation to simple hallucinations in much larger number precisely because of the subjective impression of the examinees. In contrast to that, the external impression of the hallucinations shows less representation of affirmative answers. Complex gustatory hallucinations are represented in a much larger number than the occurrence among the general population which can be explained by a wrong perception of the existing stimulus. It has been determined that 54.9% of the examinees should continue to be monitored because of gustatory hallucinations, also because of the inclusion all of the examinees with simple hallucinations and the subjective impression that they are present. Among the results there is a numerical difference noticeable related to the occurrence between the simple and complex hallucinations. Since the complex hallucinations depend on the evaluation of other people-external impression, the good thing is that the external impression is more pragmatic than the self-assessment. There were also different features of the hallucinatory experiences questioned, such as frequency, level of control and emotional reactions. The highest rate of prevalence have the intrusive thoughts (63%) while the least spread were acoustic hallucinations (25%) and visual hallucinations (29%) [23]. Several studies have found that visual hallucinations are more likely in those with Alzheimer’s disease and Lewy body pathology [24]. People who live alone are focused on their daily lives, from organizing their private lives to leaving and returning from work and maintaining a home in all aspects. They cannot rely on other people in their responsibilities, so they need more frequent controls as they become functional in the organization of their lives [25]. The prevalence of existing at least one psychotic symptom has a wide range all around the world and it goes from 0.8% to 31.4%. Psychotic symptoms signalize potential problem for public health care, independently of the complete diagnosis of psychosis because they are connected to a significant reduction in quality of health condition.

## 5. Limitations

Sample size–it would be interesting to conduct this research on few thousands of participants in different regions of Croatia.

Time constraints–conduct this research during the course od 1 or 2 years.

Lack of previous research studies on the topic in Croatia

## 6. Conclusions

The results of this research have indicated that the prevalence of hallucinations among the general population is within the European and world average is with even slightly less values. With the results of the research we can be relatively satisfied considering the socio-economic factors and cultural factors as well. While doing so, we shouldn’t neglect the war activities as well in our region of southeastern Europe three decades ago. In any case, monitoring because of hallucinations is definitely preferable because prevention is the best solution long term both for the individual and for the health care system.

## Figures and Tables

**Table 1:** Distribution of subjects according to the basic characteristics.

	Number (%) of Examinees Related to Gender			*p* *
	Women	Men	In Total	
**Marital status**				
married	103 (43)	104 (37)	207 (40)	0.003
domestic partnership	22 (9)	24 (8)	46 (9)	
Widow/widower	14 (6)	4 (1)	18 (3)	
unmarried	79 (33)	134 (47)	213 (41)	
divorced	19 (8)	18 (6)	37 (7)	
Place of residence				
City	159 (67)	182 (64)	341 (65)	0.70
Suburban area	35 (15)	42 (15)	77 (15)	
Village	43 (18)	60 (21)	103 (20)	
Where they live				
In their own apartment/house	142 (60)	136 (48)	278 (53)	0.02
In a rented apartment	35 (15)	48 (17)	83 (16)	
With their parents	60 (25)	100 (35)	160 (31)	
Level of education				
High school education	152 (64)	202 (71)	354 (68)	0.14
Higher education	50 (21)	42 (15)	92 (18)	
University graduates	35 (15)	40 (14)	75 (14)	
Working status				
Employed	163 (69)	184 (65)	347 (67)	0.06
Unemployed	74 (31)	100 (35)	174 (33)	
In total	237(100)	284 (100)	521 (100)	

* χ^2^ test.

**Table 2:** Frequency of auditory, visual, olfactory, sensory and taste hallucinations.

	Number (%)	
	No	Yes
Verification of acoustic hallucinations (Cronbach Alpha = 0.735)		
Have you ever thought you heard someone call your name, but then realized you must have been mistaken?	173 (33)	348 (67)
Have you ever heard your phone ringing, but then realized the phone hadn’t rung?	254 (49)	267 (51)
When you are falling asleep, do you ever hear strange noises? What about when you are waking up in the morning?	414 (79)	107 (21)
What about hearing music or other noises that other people around you did not seem to hear?	425 (82)	96 (18)
Have you ever had an experience where you heard things, such as loud noises, voic-es talking, or people whispering, that other people could not hear?	442 (85)	79 (15)
Have you ever had an auditory hallucination?	430 (83)	91 (17)
Have you ever been told that you are hearing things that are not real or are not really there?	459 (88)	62 (12)
Has a doctor or family member ever told you that you have had an auditory hallucina-tion?	511 (98)	10 (2)
Verification of visual hallucinations (Cronbach Alpha = 0.747)		
Have you ever had an experience in which you thought you saw something, but when you looked, nothing was there?	235 (45)	286 (55)
When you are falling asleep, do you ever see things?	430 (83)	91 (17)
Have you ever seen things move, but then realized that your eyes were playing tricks on you?	311 (60)	210 (40)
Have you ever seen things that other people could not see, such as flashes of light, or figures that were people or animals or other objects?	422 (81)	99 (19)
Have you ever had a visual hallucination?	444 (85)	77 (15)
Have you ever been told that you are seeing things that are not real or are not really there?	465 (89)	56 (11)
Has a friend, family member, or doctor ever told you that you have had a visual hallucionation?	507 (97)	14 (3)
Verification of olfactory hallucinations (Cronbach Alpha = 0.763)		
Have you ever detected an odor, such a perfume, burning, food, or trash, but could notfigure out where it was coming from?	263 (50)	258 (50)
Have you ever been near an object that seemed to have an odor it shouldn’t, or to a place that had a very unusual, mismatched odor? (such as the smell of rotting eggs when you were nowhere near a trash bin, or the smell of smoke when you couldn’t see any?)	349 (67)	172 (33)
Have you ever noticed smells that other people around you do not notice?	334 (64)	187 (36)
Have you ever smelled something but then realized that it must have been your mind playing tricks on you?	384 (74)	137 (26)
Have you ever had an olfactory, or smell, hallucination?	456 (88)	65 (12)
Have you ever been toldthat you are smelling things that are not real or are not really there?	480 (92)	41 (8)
Has a family member or doctor ever told you that youhave had an olfactory hallucination?	512 (98)	9 (2)
Verification of the tactile/cenesthetic hallucinations (Cronbach Alpha = 0.696)		
Have you ever felt something on or under your skin, but then realized you just had an itch?	274 (53)	247 (47)
Have you ever had a sensation on or under your skin that didn’t make any sense to you?	367 (70)	154 (30)
Have you ever felt things on or under your skin, like bugs crawling or something scratching or biting you, but when you looked there was nothing there?	363 (70)	158 (30)
Have you ever told a doctor about sensations on your body that concerned you but you were told nothing is wrong?	484 (93)	37 (7)
Have you ever had a tactile hallucination?	456 (88)	65 (12)
Have you ever been told that you are feeling tingling, itches or other sensations on your body that are not real?	489 (94)	32 (6)
Has a family member or doctor ever told you that you have had somatic or tactile hallucinations?	514 (99)	7 (1)
Verification of gustatory hallucionation (Cronbach Alpha = 0.658)		
Have you ever been so hungry you thought you could actually taste food in your mouth?	300 (58)	221 (42)
Have you ever eaten or drank somethingthattasted much different than you thought it should?	344 (66)	177 (34)
Have you ever suddenly had a taste in your mouth as if out of theblue, when you were not eating or drinking anything at the time?	440 (84)	81 (16)
Have you ever had a gustatory, or taste, hallucination?	469 (90)	52 (10)
Have you ever been told that you were tasting things in yourmouth that are not real, or are not really there?	510 (98)	11 (2)
Has a family member or doctor ever told you that you were having a gustatory, or taste, hallucination?	517 (99)	4 (1)

**Table 3:** Distribution of subjects according to the required control by type of hallucinations in relation to the basic characteristics of the subjects.

	Number (%) Participants Who Need Follow-up by the Type of Hallucionations				
	Auditory	Visual	Olfactory	Tactile	Gustatory
Gender					
Women	169 (71.3)	143 (60.3)	141 (59.5)	132 (55.7)	121 (51.1)
Men	219 (77.1)	195 (68.7)	177 (62.3)	162 (57)	165 (58.1)
*p* * value	0.13	0.04	0.51	0.76	0.11
Marital status					
Married/ domestic partnership	196 (77.5)	167 (66)	153 (61)	152 (60)	150 (59)
Unmarried/divorced/widow-er	192 (71.6)	171 (63.8)	165 (62)	142 (53)	136 (51)
*p* * value	0.13	0.60	0.80	0.10	0.05
Place of residence					
City	259 (76)	226 (67)	216 (64)	186 (55)	187 (55)
Suburban area	53 (69)	45 (58)	41 (53)	45 (58)	39 (51)
Village	75 (73)	66 (64)	60 (58)	62 (60)	60 (58)
*p* * value	0.38	0.41	0.20	0.57	0.60
Where they live					
In their own apartment	215 (77)	184 (66)	167 (60)	163 (59)	155 (56)
In their own house	68 (82)	61 (74)	58 (69)	46 (55)	51 (61)
With their parents	105 (66)	93 (58)	93 (58)	85 (53)	80 (50)
*p* * value	0.006	0.04	0.18	0.52	0.22
Level of education					
High school education	264 (75)	230 (65)	217 (61)	200 (57)	190 (54)
Higher education	63 (69)	54 (59)	49 (53)	51 (55)	50 (54)
University graduates	61 (81)	54 (72)	52 (69)	43 (57)	46 (61)
*p* * value	0.17	0.20	0.10	0.97	0.48
Working status					
Employed	271 (78)	229 (66)	219 (63)	198 (57)	196 (57)
Unemployed	117 (67)	109 (63)	99 (57)	96 (55)	90 (52)
*p* * value	0.006	0.42	0.16	0.66	0.29

* χ^2^ test.

**Table 4:** Frequency of simple and complex hallucination, and according to the impression of subjective and external hallucinations in relation to gender and marital status.

	Median (25–75%)					
	Gender			Marital Status		
	Women	Men	*p* *	Married/Domestic Partnership	Unmarried/Divorced/Widow/er	*p* *
Auditory hallucinations						
Simple	2 (1–2)	2 (1–2)	0.76	2 (1–2)	2 (1–2)	0.20
Complex	1.5 (1–2)	1 (1–2)	0.35	1 (1–2)	1 (1–2)	0.56
Subjective impression	2 (1–3)	2 (1–3)	0.83	2 (1–3)	2 (2–4)	0.12
External impression	1 (1–1)	1 (1–1)	0.85	1 (1–1)	1 (1–1)	0.40
Visual hallucionations						
Simple	2 (1–3)	2 (1–3)	0.30	2 (1–3)	2 (1–3)	0.64
Complex	1 (1–1)	1 (1–1)	>0.99	1 (1–1)	1 (1–1)	>0.99
Subjective impression	2 (1–3)	2 (1–3)	0.60	2 (1–3)	2 (1–3)	0.42
External impression	1 (1–1)	1 (1–1.25)	0.44	1 (1–1)	1 (1–2)	0.36
Olfactory hallucinations						
Simple	2 (1–2)	2 (1–2)	0.94	2 (1–2)	2 (1–2)	0.30
Complex	1 (1–2)	2 (1–2)	0.03	1 (1–2)	2 (1–2)	0.09
Subjective impression	2 (1.5–3)	2 (1–4)	0.63	2 (2–3)	2 (1–4)	0.95
External impressiom	1 (1–1)	1 (1–1)	0.60	1 (1–1)	1 (1–1)	0.91
Tactile hallucinations						
Simple	1 (1–1)	1 (1–1)	>0.99	1 (1–1)	1 (1–1)	>0.99
Complex	1 (1–2)	1 (1–2)	0.64	1 (1–2)	1 (1–2)	0.87
Subjective impression	2 (1–3)	2 (1–3)	0.32	2 (1–3)	2 (1–3)	0.44
External impression	1 (1–2)	1 (1–2)	0.71	1 (1–2)	1 (1–2)	0.66
Gustatory hallucinations						
Simple	1 (1–1)	1 (1–1)	>0.99	1 (1–1)	1 (1–1)	>0.99
Complex	1 (1–2)	1 (1–2)	0.59	1 (1–2)	1 (1–2)	0.44
Subjective impression	2 (1–3)	2 (1–3)	0.32	2 (1–3)	2 (1–3)	0.44
External impression	1 (1–2)	1 (1–2)	0.66	1 (1–2)	1 (1–2)	0.65

* Mann Whitney U test.

**Table 5:** Frequency of simple and complex, and according to the impression of subjective and external hallucinations in relation to the place of residence and where they live.

	Median (25–75%)							
	Place of Residence				Where They Live			
	City	Suburban Area	Village	*p* *	Own Apartment	Own House	With Parents	*p* *
Auditory hallucinations								
Simple	2 (1–2)	2 (1–2)	2 (1–2)	0.07	2 (1–2)	2 (1–2)	2 (1–2)	0.82
Complex	1 (1–2)	1 (1–2)	1 (1–2)	0.58	1 (1–2)	1 (1–2.75)	2 (1–3)	0.10
Subjective impression	2 (2–4)	1 (1–3)	2 (2–3)	0.03 †	2 (1–3)	2 (1–4)	2 (2–4)	0.75
External impression	1 (1–1)	1 (1–1)	1 (1–2)	0.11	1 (1–1)	1 (1–1)	1 (1–1)	0.51
Visual hallucinations								
Simple	2 (1–3)	2 (1–2)	2 (1–3)	0.28	2 (1–3)	2 (1–3)	2 (1–2)	0.88
Complex	1 (1–1)	1 (1–1)	1 (1–1)	>0.99	1 (1–1)	1 (1–1)	1 (1–1)	>0.99
Subjective impression	2 (1–3)	2 (1–2)	2 (1–3)	0.23	2 (1–3)	2 (1–3)	2 (1–3)	0.69
External impression	1 (1–1)	1 (1–1.5)	1 (1–2)	0.84	1 (1–1)	1 (1–1.25)	1 (1–1)	0.99
Olfactory hallucinations								
Simple	2 (1–2)	2 (1–2)	2 (1–2)	0.84	2 (1–2)	2 (1–2)	2 (1–2)	0.42
Complex	2 (1–2)	1 (1–2)	2 (1–2)	0.74	1 (1–2)	2 (1–2)	2 (1–2)	0.40
Subjective impression	3 (2–4)	2 (1–3.5)	2 (1–3)	0.19	2 (2–3)	3 (1–4)	2 (1–4)	0.77
External impression	1 (1–1)	1 (1–1)	1 (1–1)	0.64	1 (1–1)	1 (1–1)	1 (1–1)	0.85
Tactile hallucinations								
Simple	1 (1–1)	1 (1–1)	1 (1–1)	>0.99	1 (1–1)	1 (1–1)	1 (1–1)	>0.99
Complex	1 (1–2)	1 (1–2)	1 (1–2)	0.43	1 (1–2)	2 (1–2)	1 (1–2)	0.29
Subjective impression	2 (1–3)	2 (1.3–2.8)	2 (1–3)	0.26	2 (1–3)	2 (1–3)	2 (1–2)	0.14
External impression	1 (1–1.8)	1 (1–2)	1 (1–2)	0.21	1 (1–2)	1 (1–1.5)	1 (1–2)	0.44
Gustatory hallucinations								
Simple	1 (1–1)	1 (1–1)	1 (1–1)	>0.99	1 (1–1)	1 (1–1)	1 (1–1)	>0.99
Complex	1 (1–2)	2 (1–2)	1 (1–2)	0.11	1 (1–2)	1 (1–2)	1 (1–2)	0.34
Subjective impression	2 (1–3)	2 (1.3–2.8)	2 (1–3)	0.26	2 (1–3)	2 (1–3)	2 (1–2)	0.14
Eternal impression	1 (1–1)	1 (1–2)	1 (1–2)	0.08	1 (1–2)	1 (1–1)	1 (1–2)	0.58

* Kruskal Wallis test (Post hoc Conover). † at the level of *p* < 0.05 there are significant differences between city vs. suburban area.

**Table 6:** Frequency of simple and complex, and according to the impression of subjective and external hallucinations in relation to the level of education and employment status.

	Median (25–75%)						
	Level of Education				Working Status		
	High School Education	Higher Education	University Graduates	*p* *	Unemployed	Employed	*p* *
Auditory hallucinations							
Simple	2 (1–2)	2 (1–2)	2 (1–2.5)	0.44	2 (1–2)	2 (1–2)	0.54
Complex	1 (1–2)	1 (1–2)	2 (1–2.3)	0.15	1 (1–2)	1 (1–3)	0.20
Subjective impression	2 (2–3)	2 (1–3)	2 (1.5–4)	0.35	2 (1–3)	2 (2–4)	0.35
External impression	1 (1–1)	1 (1–1.25)	1 (1–1)	0.82	1 (1–1)	1 (1–1)	>0.99
Visual hallucinations							
Simple	2 (1–3)	2 (1–2)	2 (1–3)	0.79	2 (1–3)	2 (1–3)	0.23
Complex	1 (1–1)	1 (1–1)	1 (1–1)	>0.99	1 (1–1)	1 (1–1)	>0.99
Subjective impression	2 (1–3)	2 (1–3)	2 (1–3)	0.52	2 (1–3)	2 (1–3)	0.40
External impression	1 (1–1.3)	1 (1–2)	1 (1–1)	0.18	1 (1–1)	1 (1–2)	0.21
Olfactory hallucinations							
Simple	2 (1–2)	2 (1–2)	2 (1–2)	0.98	2 (1–2)	2 (1–2)	0.85
Complex	1.5 (1–2)	1 (1–2)	2 (1–2)	0.87	1 (1–2)	2 (1–2)	0.37
Subjective impression	2 (1–4)	2 (1–3)	3 (2–4)	0.33	3 (2–3)	2 (1–4)	0.48
External impression	1 (1–1)	1 (1–2)	1 (1–1.5)	0.37	1 (1–1)	1 (1–1)	0.87
Tactile hallucinations							
Simple	1 (1–1)	1 (1–1)	1 (1–1)	>0.99	1 (1–1)	1 (1–1)	>0.99
Complex	1 (1–2)	1 (1–2)	1 (1–2)	0.86	1 (1–2)	1 (1–2)	>0.99
Subjective impression	2 (1–3)	2 (1–3)	2 (1–3)	0.59	2 (1–3)	2 (1–3)	0.26
External impression	1 (1–2)	1 (1–1)	2 (1–2)	0.23	1 (1–2)	1 (1–2)	0.71
Gustatory hallucinations							
Simple	1 (1–1)	1 (1–1)	1 (1–1)	>0.99	1 (1–1)	1 (1–1)	>0.99
Complex	1 (1–2)	1 (1–2)	1 (1–2)	0.50	1 (1–2)	1 (1–2)	0.33
Subjective impression	2 (1–3)	2 (1–3)	2 (1–3)	0.59	2 (1–3)	2 (1–3)	0.26
External impression	1 (1–2)	1 (1–1.3)	1 (1–2)	0.34	1 (1–2)	1 (1–2)	0.89

* Kruskal Wallis test (Post hoc Conover).

## Data Availability

Not applicable.
